# Unusual Presentation of Mediastinal Neurogenic Tumours

**DOI:** 10.1155/2013/414260

**Published:** 2013-05-08

**Authors:** Giampiero Negri, Alessandro Bandiera, Angelo Carretta, Armando Puglisi, Carlo Mandelli, Paola Ciriaco, Piero Zannini

**Affiliations:** ^1^Department of Thoracic Surgery, Scientific Institute H San Raffaele, Via Olgettina 60, 20132 Milan, Italy; ^2^Department of Neurosurgery, Scientific Institute H San Raffaele, Via Olgettina 60, 20132 Milan, Italy

## Abstract

Mediastinal neurogenic tumours generally arise as single benign lesions and their typical location is the costovertebral sulcus. In about 10% of cases mediastinal neurogenic tumours may extend to the spinal canal; occasionally they may extend to the cervical region and, more rarely, may be multiple or associated with other synchronous mediastinal lesions. The treatment of choice is surgical resection. This report describes three cases of unusual presentation of mediastinal benign schwannomas successfully treated at our Hospital. In the first case multiple simultaneous paravertebral lesions were resected through a posterior approach. In the second case a tumour of the posterior mediastinum extending to the cervical region was excised through a one-stage combined supraclavicular incision followed by left mini-invasive video-assisted thoracoscopic surgical techniques. The third case describes a patient with a posterior neurogenic mediastinal tumour with a synchronous parathyroid adenoma of the anterior mediastinum, which were both successfully resected by video-assisted thoracoscopic surgery.

## 1. Introduction

Mediastinal schwannomas are the most common neurogenic tumours, which generally involve the posterior mediastinum. These tumours usually arise as single benign lesions in the costovertebral sulcus [1] and only rarely as multiple lesions. In about 10% of cases mediastinal schwannomas may extend to the spinal canal (dumbbell tumours) [2]; occasionally they may also extend to the cervical region or, even more rarely, may be associated with other synchronous mediastinal lesions with a different histology. 

Surgical resection is the treatment of choice for benign schwannomas as well as for other posterior mediastinal neurogenic tumours. Mini-invasive video-assisted thoracoscopic surgery (VATS) has progressively become the gold standard approach to these tumours, as it has proven to be a safe and reliable approach, with excellent surgical results and with less morbidity as compared to open surgery [3]. However, in specific cases when the lesions extend to the cervical region or involve the spinal canal cooperation with neurosurgeons is mandatory in order to avoid neurological lesions. We report three cases of benign schwannomas of the posterior mediastinum with an unusual presentation. In one patient simultaneous multiple paravertebral lesions were observed; in a second patient the tumour originating from the posterior mediastinum extended to the cervical region. A third patient presented with a posterior neurogenic mediastinal tumour with a synchronous parathyroid adenoma of the anterior mediastinum, both of which were successfully resected by VATS.

## 2. Case Report

**Case 1**

**Figure 1:**
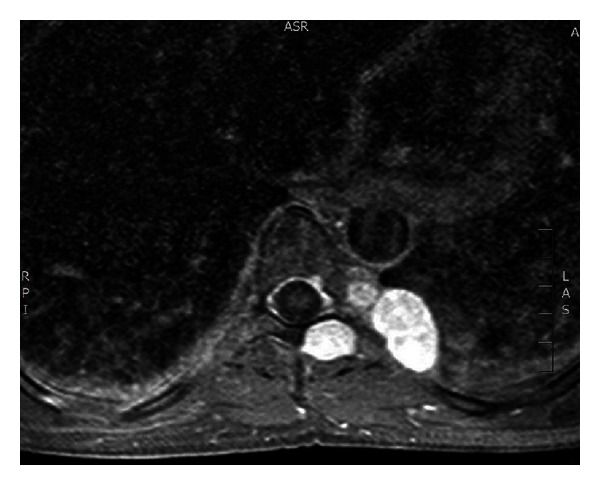
Magnetic resonance imaging (MRI) showing multiple paravertebral schwannomas.

A 41-year-old woman underwent radiological assessment at our hospital following the acute onset of back pain. A spine magnetic resonance imaging (MRI) revealed multiple paravertebral oval-shaped tumoural lesions. The largest tumour (2 × 3.5 cm in diameter) originated in the left costovertebral sulcus and laid on the eighth rib. Medially to this tumour another similar lesion involved the eighth left intervertebral foramen. Two smaller lesions were detected in the contiguous vertebral canal and another one was identified in the muscular tissue outside the vertebral lamina (Figure 1). This last lesion was excised first through a posterior vertical midline incision extending from T7 to T11. A left eighth hemilaminectomy was then performed to remove the two lesions in the vertebral canal; finally the two mediastinal tumours were excised extrapleurally through a left eighth costotransversectomy. The patient had an uneventful recovery without neurological damage. Back pain disappeared after surgical resection. At pathologic examination all lesions were benign schwannomas.

The patient is alive with no evidence of relapse 24 months after the operation.

**Case 2**

**Figure 2:**
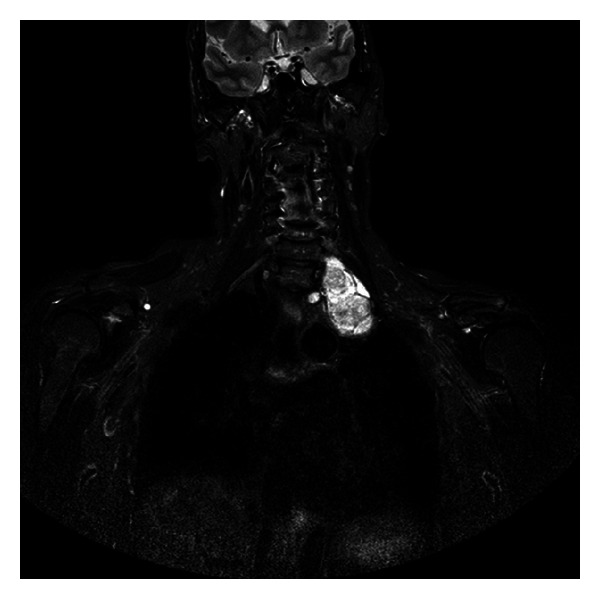
Magnetic resonance imaging (MRI) showing a left posterior mediastinal schwannoma extending to the cervical region through the thoracic inlet.

A 58-year-old woman was admitted to our Hospital after the incidental finding of a left apical lesion at a routine chest X-ray. Computed tomography (CT) scan showed a well-encapsulated superior posterior mediastinal tumour, 6.5 × 4.5 cm in diameter, extending to the cervical region. On MRI the lesion was seen to be close to the brachial plexus without any sign of infiltration (Figure 2). Surgical approach was a one-stage combined supraclavicular incision followed by left VATS. The supraclavicular incision allowed a safe and accurate exposure and mobilization of the cervical portion of the tumour from the superior and median trunk of the brachial plexus and from the subclavian artery. The patient was then repositioned on the operating table on right lateral decubitus and dissection of the tumour from the inferior trunk of the brachial plexus and its en-bloc removal from the chest were performed through a left mini-invasive video-assisted thoracic approach with a 6 cm auxiliary thoracotomy. The patient had an uneventful recovery with only a transient mild strength deficit in the left hand. Pathologic examination revealed a benign schwannoma.

The patient is alive with no evidence of relapse 52 months after the operation.

**Case 3**

**Figure 3:**
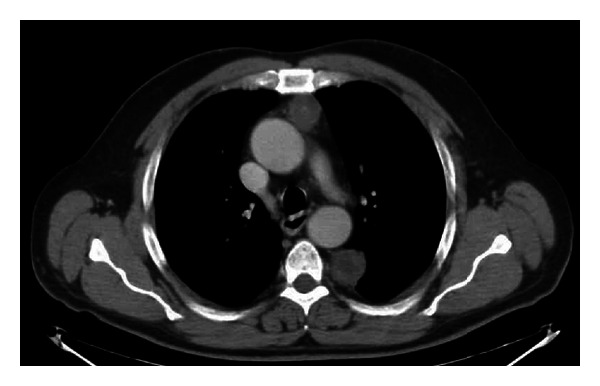
Computerized tomography (CT) scan showing a left posterior mediastinal schwannoma associated with an anterior parathyroid adenoma.

A 63-year-old man was admitted to our hospital after the incidental finding of a left paravertebral lesion at a routine chest X-ray. CT scan demonstrated the presence of a left posterior mediastinal tumour (7 cm in diameter) at the level of T6–T8 associated with an anterior well-encapsulated mediastinal mass (2.5 cm in diameter) (Figure 3). Both mediastinal lesions were removed through a single-stage three-portal VATS procedure. All the ports were positioned in the middle axillary line. The patient had an uneventful postoperative recovery. Pathologic examination of the posterior tumour revealed a benign schwannoma while the anterior mediastinal lesion resulted in a parathyroid adenoma.

The patient is alive with no evidence of relapse 24 months after the operation.

## 3. Discussion

Mediastinal schwannomas are neurogenic tumours which generally arise in the posterior costovertebral sulcus. In adults these lesions are benign in most of the cases and have an intraspinal extension in about 10% of patients [2, 4]. 

Mediastinal schwannomas generally present as single lesions. Only rarely do they have a multifocal presentation. Chen et al. [5] described successful thoracoscopic resection of multiple schwannomas arising from a single intercostal nerve in the posterior chest wall. In the first case we described, multiple contiguous schwannomas were observed both in the posterior mediastinum and inside the vertebral canal, with a further schwannoma outside the vertebral lamina probably arising from the sensitive nerve of the corresponding nervous root. All the lesions were successfully resected through a posterior approach. Because of the limited size of the mediastinal tumours they could be removed extrapleurally without necessitating a second thoracic procedure.

Occasionally mediastinal neurogenic tumours extend to the cervical region. Yamaguchi et al. [6] and Akashi et al. [7] described the surgical excision of cervicomediastinal neurogenic tumours by means of a preliminary VATS to dissect the tumour from the mediastinal tissue followed by a supraclavicular incision for the complete dissection and the removal of the tumour. In our case (Case 2) a supraclavicular approach was chosen to safely manipulate the schwannoma away from the nervous and vascular structures of the thoracic inlet and to facilitate dissection. Once the tumour was mobilized from the neck a mini-invasive thoracic procedure was performed to dissect it from the upper mediastinum and to remove it.

Posterior neurogenic tumours may at times be associated with other mediastinal lesions localized in the anterior mediastinum. This is a very rare event. Chon et al. [8] described a successful single-stage robotic-assisted thoracoscopic excision of a posterior schwannoma and an anterior thymic cyst. We used a single-stage three-portal VATS procedure to resect a posterior schwannoma and an anterior parathyroid adenoma (Case 3). All the thoracoscopic ports were positioned in the middle axillary line in order to dissect and remove both lesions in the posterior and in the anterior mediastina.

More rarely, mediastinal schwannomas originate from vagus or phrenic nerve and should be removed through a nerve-sparing tumour technique [9].

In conclusion, considering that mediastinal schwannomas are generally benign tumours, all efforts should be directed towards their surgical resection with mini-invasive approaches even when they arise as multiple simultaneous lesions or in unusual locations. For those lesions extending in the cervical region or involving the spinal canal a multidisciplinary team including thoracic surgeons and neurosurgeons allows their resection with a safe single-stage procedure.
